# Papillary Thyroid Carcinoma Originating in a Male With Thyroglossal Duct Cyst: Case Presentation and Literature Review

**DOI:** 10.1002/ccr3.72604

**Published:** 2026-04-23

**Authors:** Nasim Abukaresh, Hossam Salameh, Tala Dweik, Anan Dawadi, Haya Taha, Sohaib Salahat, Ahmed Aljodi

**Affiliations:** ^1^ Palestine Polytechnic University Hebron Palestine; ^2^ Department of Medicine, Faculty of Medicine and Health Sciences An‐Najah National University Nablus Palestine; ^3^ Al‐Quds University Jerusalem Palestine; ^4^ Bogomolets National Medical University Kyiv Ukraine; ^5^ Radiology Department Hebron Governmental Hospital Hebron Palestine; ^6^ Department of General Surgery Hebron Governmental Hospital Hebron Palestine

**Keywords:** neck mass, papillary thyroid carcinoma, Sistrunk procedure, thyroglossal duct cyst, thyroglossal duct cyst carcinoma

## Abstract

Papillary carcinoma arising in a thyroglossal duct cyst is rare and often indistinguishable from benign disease. We report a male patient with progressive cyst changes leading to diagnosis and successful Sistrunk‐based management, highlighting the need for close, appropriate imaging and individualized treatment in midline neck masses.

## 1 Introduction

Congenital midline neck masses represent common developmental anomalies that can manifest at birth or later in life, with a broad differential diagnosis [1]. During early embryogenesis, the thyroid gland descends from the foramen cecum to a location beneath the thyroid cartilage, leaving behind an epithelial tract known as the thyroglossal duct. This tract typically involutes between the fifth and tenth weeks of gestation; failure of complete involution can result in the formation of a TGDC, which may contain cystic, ductal, or ectopic thyroid tissue [1, 2].

TGDCs are the most common congenital thyroid gland anomalies and account for approximately 7% of midline neck masses in adults [3]. Malignant transformation within a TGDC is rare, occurring in about 0.7%–1.5% of cases. Papillary thyroid carcinoma (PTC) is the predominant histological type, while squamous cell carcinoma (SCC) accounts for a minority. Clinically, TGDC carcinoma may present as a rapidly enlarging or asymptomatic midline neck mass, often indistinguishable from a benign cyst before surgical excision [2].

This report describes the clinical presentation, diagnostic evaluation, surgical management, and follow‐up of a 47‐year‐old man diagnosed with PTC arising in a TGDC.

## 2 Case History/ Examination

A 47‐year‐old man presented to our surgical clinic with a painless midline neck mass that had been present for 1 year without any noticeable change in size. He denied fever, hypo or hyperthyroidism symptoms, compressive complaints like dysphagia, dyspnea, or dysphonia, and he had no previous radiation exposure or a personal or family history of thyroid disease. Clinical examination revealed a well‐defined, hard‐elastic, non‐tender mass measuring approximately 0.5 × 0.5 cm, located just to the right of midline beneath the hyoid bone. The mass moved with tongue protrusion, and no cervical lymphadenopathy was detected. Thyroid function tests and routine blood parameters were within reference ranges.

## 3 Differential Diagnosis, Investigations, and Treatment

Initial neck ultrasonography demonstrated a 0.7 × 0.5 cm right‐paramedian cystic lesion with thin internal septations and posterior acoustic enhancement, without mural nodules or vascularity, and with a sonographically normal thyroid gland. These findings were consistent with a benign TGDC. However, a follow‐up ultrasound several months later demonstrated interval change: the lesion had enlarged to 1.6 cm. It evolved into a mixed solid–cystic mass fixed at the level of the hyoid bone, raising concern for malignant transformation despite the absence of cervical lymphadenopathy.

**FIGURE 1:**
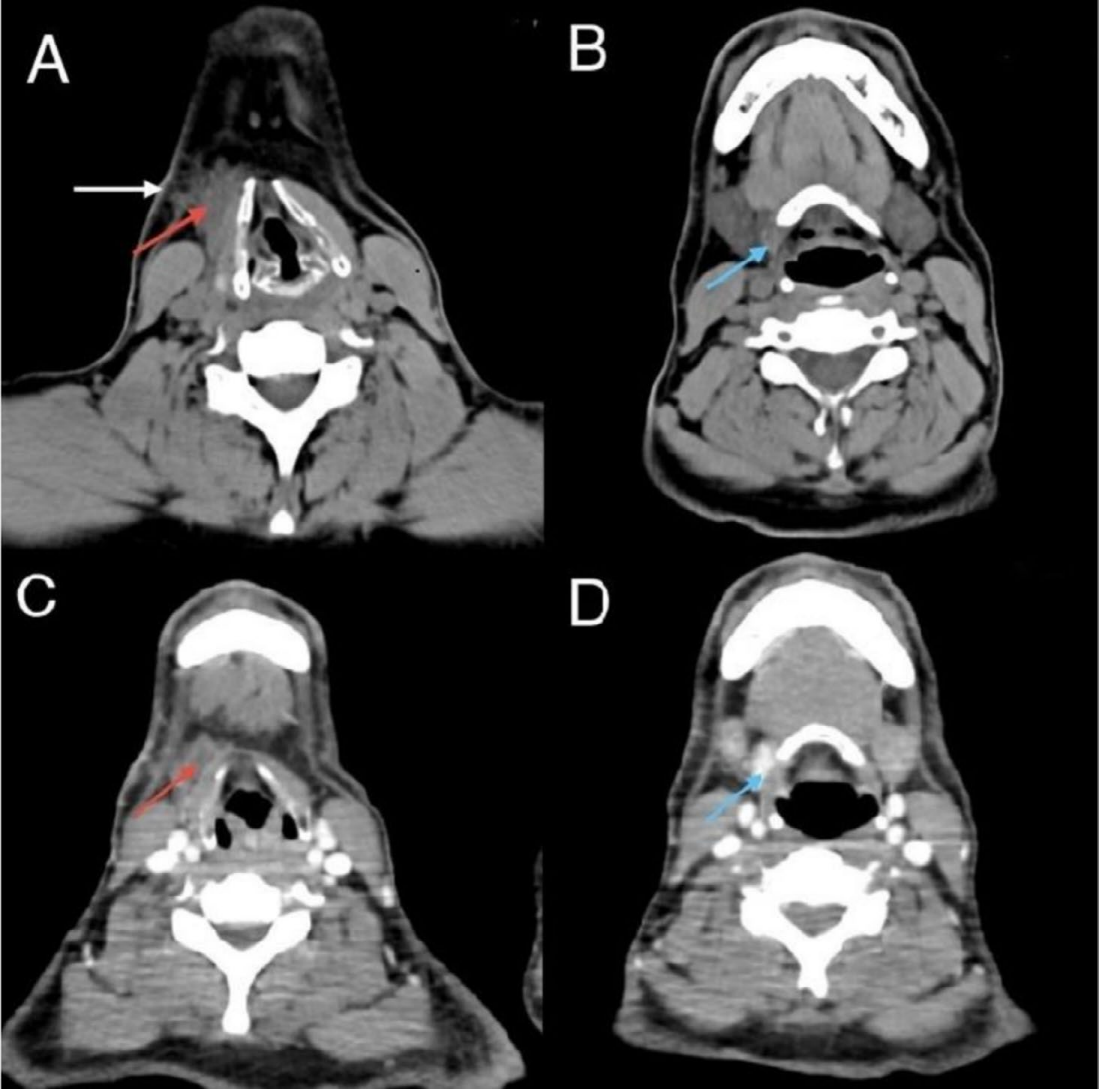
Axial neck CT images without iodinated contrast (A, B) and with iodinated contrast (C, D) showing a paramedline infrahyoid cystic lesion (red arrow) with a hyperdense solid enhancing component (blue arrow) surrounded by fat stranding (white arrow). A neck CT scan revealed a paramedline infrahyoid lesion embedded in the right infrahyoid muscle. It had cystic and solid components, no calcifications, and was surrounded by fat stranding. The presumed diagnosis was an infected Thyroglossal duct cyst with ectopic thyroid tissues.

To further assess local extension, contrast‐enhanced CT of the neck and chest was performed. This showed a well‐defined 1.5 × 0.7 cm lesion adjacent to but not invading the hyoid bone or strap muscles, and with density similar to thyroid tissue (shown in Figure 1). No suspicious lymph nodes or intrathyroidal lesions were seen. The multidisciplinary team discussion addressed management options, including total thyroidectomy versus limited excision. The team was composed of a surgical oncologist, a medical oncologist, an endocrinologist, and a radiologist.

**FIGURE 2:**
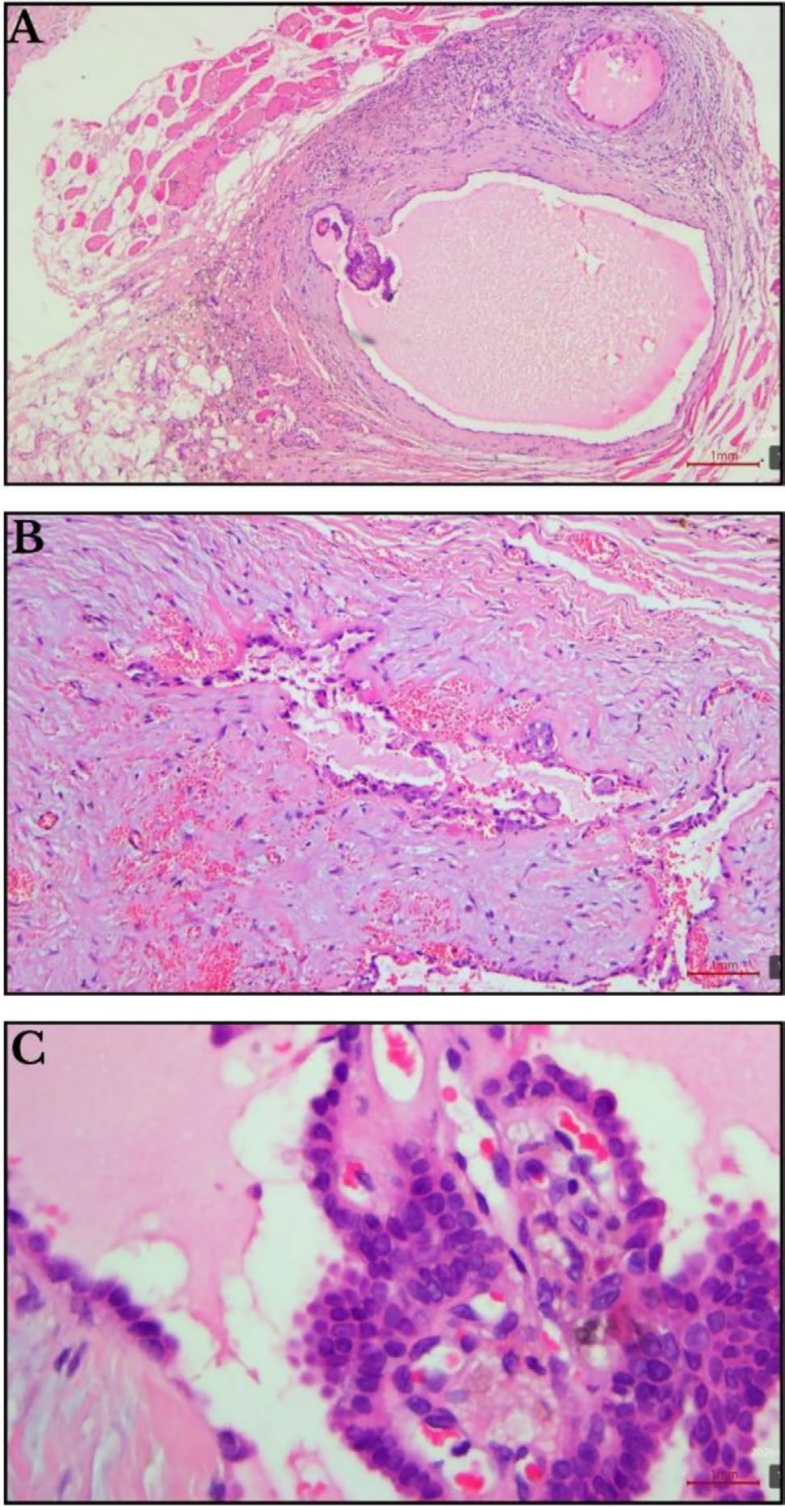
(A) Some epithelial cells with papillary architecture and cystic spaces are noted within fibrofatty and muscle tissue. This wide view shows a large circular “cystic” space and organized papillary structures, with clear skeletal muscle bundles (bright pink) visible at the periphery. (B) Groups of epithelial cells within a fibrous and myxoid stroma. (C) The epithelial cells show nuclear elongation, overlapping, and some clearing. This high‐power H&E view focuses on the nuclei. With characteristic “clearing” (Orphan Annie eye appearance) and crowding/overlapping.

**FIGURE 3:**
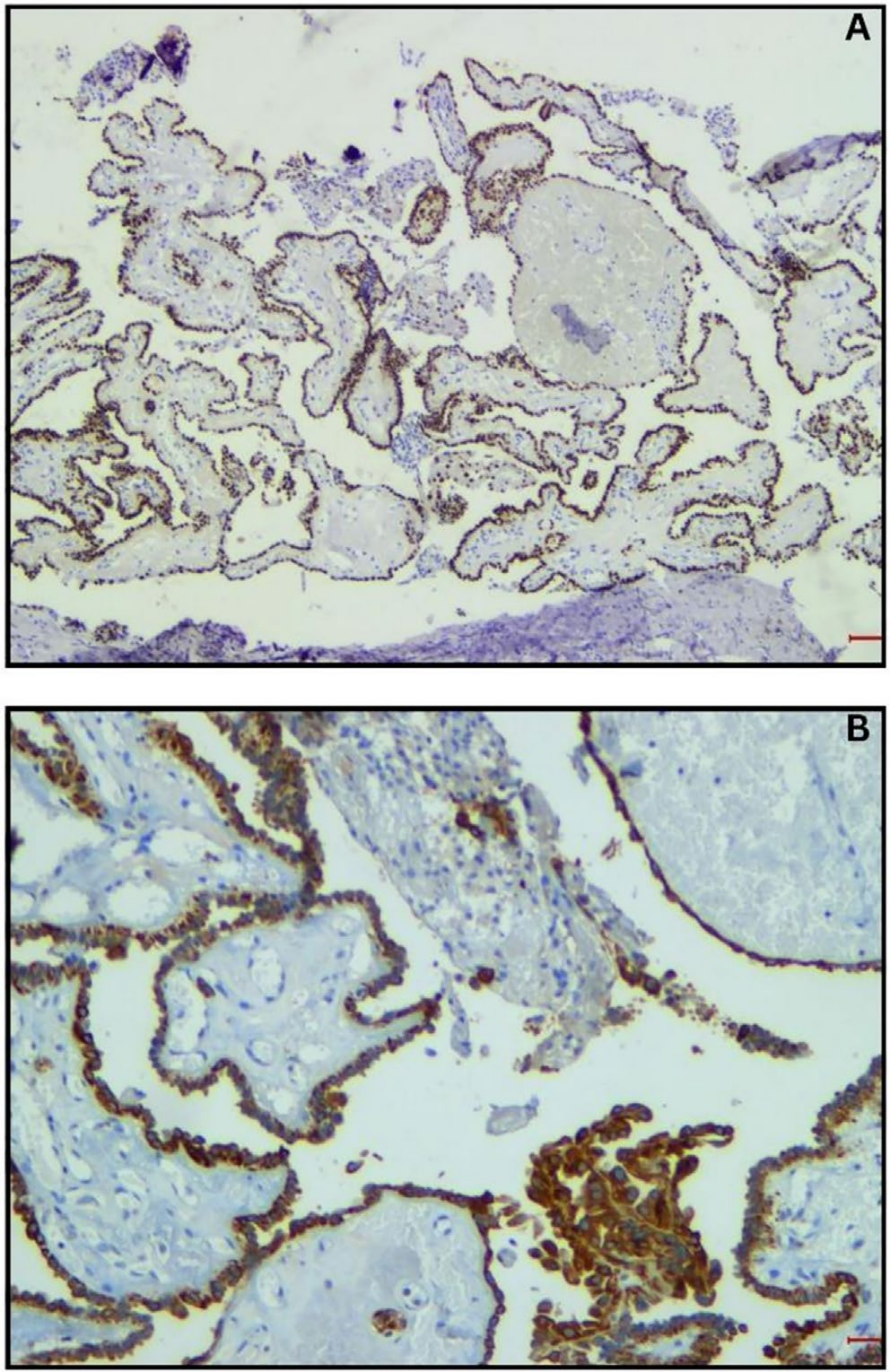
(A) The neoplastic cells are diffusely positive for pan‐CK IHC stain. (B) Positive for TTF1 IHC stain. This high‐magnification IHC slide shows distinct nuclear brown staining. Tumor cells show nuclear positivity for TTF‐1.

Given the absence of radiologic thyroid involvement, patient preference, and low‐risk histologic features, the patient decided against total thyroidectomy. A wide local excision following the Sistrunk principles was performed, removing a 1.2 cm papillary carcinoma confined to the cyst without lymphovascular invasion or skin involvement, which revealed PTC arising within the TGDC. Histopathology demonstrated classic papillary architecture with nuclear clearing and psammoma bodies (shown in Figure 2), and diffuse cytokeratin‐pan positivity (shown in Figure 3). Margin assessment was limited due to specimen fragmentation, but no residual disease was suspected.

Postoperatively, the patient recovered uneventfully and was given 100 μg/day of thyroxine as part of treatment for thyroid hormone suppression. Follow‐up evaluation with neck ultrasonography and radionuclide scanning showed no residual mass or lymphadenopathy. Thyroid hormone levels (TSH, T3, T4) remained within therapeutic target ranges, and no clinical or radiologic recurrence was detected during subsequent visits after 2 years.

## 4 Conclusion

Papillary carcinoma arising in a TGDC is an uncommon malignancy that often mimics benign disease and is therefore frequently diagnosed only after surgical excision. Careful clinical assessment, appropriate imaging, and histopathological evaluation are essential for accurate diagnosis and risk stratification. Management should be individualized based on tumor characteristics, patient factors, and the presence of suspected thyroid or nodal involvement. Our case highlights the importance of close follow‐up and demonstrates that, in selected low‐risk patients, Sistrunk‐based excision with thyroid hormone suppression can achieve excellent outcomes.

## 5 Discussion

TGDCs are the most common congenital anomaly of the thyroid; however, thyroglossal duct cyst carcinomas (TGDCCs) are extremely rare, with 90% originating from thyroid remnants. Most cases of cancer are papillary, while fewer than 5% are SCCs [1]. Although papillary carcinoma is the most common type of thyroid cancer found in TGDC remnants, clinicians must remain vigilant due to the heterogeneity of thyroid malignancies. Gambardella et al. reported a rare medullary thyroid carcinoma lacking classical biochemical markers, illustrating that thyroid malignancies may occasionally deviate from expected histologic or immunohistochemical profiles. While medullary carcinoma is not expected in TGDCs, because there are no parafollicular cells, this example highlights the importance of thorough histopathological evaluation and appropriate marker interpretation in unusual thyroid malignancies to ensure accurate diagnosis [4]. TGDCCs occur most frequently in the fourth decade of life and are more common in women than men (female: male ratio 3:2) [5]. They typically present as a rapidly enlarging, tender neck mass, although some cases remain asymptomatic [5]. The etiology of TGDCCs is not definitively known.

Two main theories explain how carcinomas arise within thyroglossal duct cysts. The de novo theory suggests the tumor arises directly from ectopic thyroid tissue within the cyst, supported by the fact that ectopic tissue is found in 62% of cases and by the absence of medullary carcinoma, which originates from parafollicular cells. The metastatic theory proposes that TGD carcinoma represents metastasis from an occult primary thyroid tumor. But this theory is less likely, as multicentricity and multifocal growths are common in papillary thyroid carcinomas [6, 7, 8]. One previous report proposed that SCC may be the only true primary tumor of the thyroglossal duct, as most other cancers arise from ectopic thyroid tissue [9]. Although rare, SCC carries a much worse prognosis than papillary carcinoma, with reported mortality rates of 30%–40% [6, 7, 8].

**FIGURE 4:**
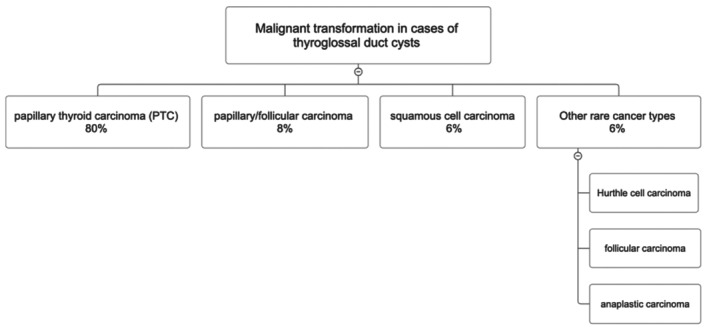
The type of malignant transformation in cases of thyroglossal duct cysts.

Because the clinical presentation closely resembles that of benign TGDCs, most malignant cases are identified only after surgical excision, with the final diagnosis depending on histopathological evaluation [5, 10]. The overall risk of malignant transformation is approximately 1%, and histological classification is outlined in Figure 4 [3]. Cervical lymph node metastasis occurs in 7%–15% of cases, which is lower than in PTC arising in the thyroid gland [5]. Mortality associated with TGDCCs remains extremely low [5]. The decision regarding the extent of surgery and lymph node management remains a subject of debate. Our case was treated with an Sistrunk‐based excision, but the broader literature on PTC emphasizes risk‐focused management. In most cases, selective neck dissection is preferred over routine neck dissection unless there is evidence of lymph node metastasis [11]. In this way, radical procedures can be avoided while maintaining oncological safety.

Neck ultrasound is recommended as the first‐line imaging modality for evaluating TGDCs and confirming the presence of a normally positioned thyroid gland. MRI and CT scans provide further characterization and can demonstrate a solid nodule within the cyst, wall thickening, calcifications, or irregular margins [5]. In our patient, both ultrasound and CT revealed features suggestive of malignancy, including a solid component, calcifications, and marked vascularity. Although the diagnosis is typically made postoperatively by histologic examination, there is currently no consensus on optimal management strategies for TGDCCs [5]. Although clinical assessment and imaging remain fundamental, emerging evidence suggests that transcutaneous laryngeal ultrasonography is an effective and safe method for assessing vocal cord mobility preoperatively. Postoperative recurrent laryngeal nerve palsies are one of the most common causes of medicolegal claims. According to a prospective multicenter study, this ultrasound‐based technique offers reliable preoperative assessments of vocal cord function, thus supporting nerve integrity evaluation before thyroid surgery [12].

Conservative management of PTC is reserved for low‐risk patients: female, younger than 40 years, with low‐grade tumors < 1 cm and no evidence of capsular invasion. For pure SCC arising in a TGDC, the Sistrunk procedure alone is considered adequate. Conversely, in cases of differentiated thyroid carcinoma within a TGDC, total thyroidectomy is recommended regardless of clinical or radiologic thyroid involvement. For tumors > 1 cm, those invading the cyst wall, or when suspicious foci are present in the thyroid gland, total thyroidectomy followed by radioiodine (I^131^) ablation and TSH suppression is the most commonly advocated approach. Thyroidectomy facilitates staging and enhances detection of metastasis or recurrence, particularly given the multicentric potential of papillary carcinoma. Regarding cervical lymph nodes, even non‐palpable nodes warrant intraoperative frozen section evaluation. Radical or modified neck dissection is indicated only when lymph node metastasis is confirmed [1, 13].

## Author Contributions

**Nasim Abukaresh:** writing – original draft, writing – review and editing. **Hossam Salameh:** visualization, writing – original draft, writing – review and editing. **Tala Dweik:** writing – original draft, writing – review and editing. **Anan Dawadi:** writing – original draft, writing – review and editing. **Haya Taha:** writing – original draft, writing – review and editing. **Sohaib Salahat:** writing – original draft, writing – review and editing. **Ahmed Aljodi:** supervision.

## Funding

The authors have nothing to report.

## Disclosure

Health and Safety: Authors confirm that all mandatory laboratory health and safety procedures have been complied with in the course of conducting any experimental work reported in this paper.

## Ethics Statement

All procedures performed in this report involving human participants were in accordance with the ethical standards of the institutional, national research committee, and with the 1964 Helsinki declaration and its later amendments or comparable ethical standards.

## Consent

Authors obtained verbal and written informed consent from the patient regarding this case and any accompanying images. A copy of the written consent is available for review by the Editor‐in‐Chief of this journal on request.

## Data Availability

Data sharing not applicable to this article as no datasets were generated or analysed during the current study.
